# Bad to the Bone: The Effects of Therapeutic Glucocorticoids on Osteoblasts and Osteocytes

**DOI:** 10.3389/fendo.2022.835720

**Published:** 2022-03-31

**Authors:** Manuel Gado, Ulrike Baschant, Lorenz C. Hofbauer, Holger Henneicke

**Affiliations:** ^1^ Center for Regenerative Therapies TU Dresden, Technische Universität Dresden, Dresden, Germany; ^2^ Department of Medicine III, University Hospital Carl Gustav Carus, Technische Universität Dresden, Dresden, Germany; ^3^ Center for Healthy Aging, University Hospital Carl Gustav Carus, Technische Universität Dresden, Dresden, Germany

**Keywords:** glucocorticoids, osteoblasts, osteocytes, glucocorticoid-induced osteoporosis (GIO), anti-resorptive treatment, osteo-anabolic treatment

## Abstract

Despite the continued development of specialized immunosuppressive therapies in the form of monoclonal antibodies, glucocorticoids remain a mainstay in the treatment of rheumatological and auto-inflammatory disorders. Therapeutic glucocorticoids are unmatched in the breadth of their immunosuppressive properties and deliver their anti-inflammatory effects at unparalleled speed. However, long-term exposure to therapeutic doses of glucocorticoids decreases bone mass and increases the risk of fractures – particularly in the spine – thus limiting their clinical use. Due to the abundant expression of glucocorticoid receptors across all skeletal cell populations and their respective progenitors, therapeutic glucocorticoids affect skeletal quality through a plethora of cellular targets and molecular mechanisms. However, recent evidence from rodent studies, supported by clinical data, highlights the considerable role of cells of the osteoblast lineage in the pathogenesis of glucocorticoid-induced osteoporosis: it is now appreciated that cells of the osteoblast lineage are key targets of therapeutic glucocorticoids and have an outsized role in mediating their undesirable skeletal effects. As part of this article, we review the molecular mechanisms underpinning the detrimental effects of supraphysiological levels of glucocorticoids on cells of the osteoblast lineage including osteocytes and highlight the clinical implications of recent discoveries in the field.

## Introduction

Harvey Cushing first described the development of ‘osteoporosis of the skeleton’ in the spine of patients suffering from endogenous hypercortisolism 90 years ago (1). Two decades later, clinicians observed the same phenomenon in patients receiving synthetic glucocorticoids (GCs) (2). GC-induced osteoporosis (GIO) is considered the third most common condition of pathological bone loss following post-menopause and aging, and is the most frequent cause of secondary osteoporosis. For instance, in the Global Longitudinal Study of Osteoporosis in Women (GLOW), about 2.7-4.6% of women from 10 different countries received treatment with GCs (3). Although a considerable proportion of GC-induced fractures remain asymptomatic and thus difficult to detect, exposure to exogenous GCs has been linked to a high incidence of fractures, particularly in the spine. A rapid reduction in bone mineral density (BMD) is generally observed as early as 3-6 months after initiation of GC treatment and persists during continued GC exposure (4–9). Aside from the spine, typically locations of GC-induced fractures include the ribs and pelvis (8, 10–12), indicating that sites rich in trabecular bone are more affected than the cortical structures (10). Interestingly, some studies observed a rapid development of fractures in patients receiving GCs, even before any detectable decreases in the bone mineral density (9, 13, 14), suggesting that not just bone mass but also bone quality is compromised in the presence of supra-physiological levels of GCs (**Box 1**).

Several molecular mechanisms underlying GIO have been identified through *in vivo* and *in vitro* studies. Overall, the effects of excess GCs in the skeleton are complex owing to the multifaceted nature of interactions between local and systemic factors. Generally, GCs act *via* the glucocorticoid receptor (GR), which is ubiquitously expressed in all skeletal cell types. The molecular nature of GC-GR interactions and their interplay with target cells are manifold and complex. Briefly, upon ligand binding the GR translocates to the nucleus where it either acts as a dimer by binding directly to the DNA in the promotor region of target genes or it may act as a monomer by interfering with other transcription factors such as activator protein 1 (AP-1) and nuclear factor kappa-light-chain-enhancer of activated B cells (NF-κB). A detailed review of the molecular action of the GC-GR complex is provided by Hartmann et al. (18) or Vandewalle et al. (19).

The skeletal effects of therapeutic GC use have to be separated from the role of physiological GCs in the skeleton. Physiological concentrations of GCs are critically required for differentiation of stromal progenitors towards the osteoblast lineage – and away from adipocytes – (20, 21) and thus support bone formation (22) and the accrual of bone mass (23–25). Overall, physiological concentrations of GCs exert anabolic effects throughout the skeleton particularly during growth, whereas supraphysiological (or therapeutic) levels of GCs result in loss of bone mass and quality (26, 27). Early studies on GIO have described several extra-skeletal effects, which may mechanistically underpin GC-induced bone loss, such as i) a dysregulation of calcium homeostasis through decreased intestinal calcium absorption and increased renal calcium clearance; ii) a reduction in the growth hormone/insulin-like growth factor axis; iii) alteration in gonadal steroid hormones; or iv) the potential development of secondary hyperparathyroidism. Also, the catabolic effects of GCs on skeletal muscle have been marked as a contributor to increased fracture risk *via* increased incidence of falls secondary to muscle weakness (28–30). Interestingly, over the last two decades, advances in mouse genetics have enabled the detailed characterization of the mechanisms of GC-induced bone loss. This led to the discovery that the direct effects of supra-physiological levels of GCs on bone cells represent a significant part of the pathogenesis of GIO. Generally, the pathogenesis of GIO is characterized by two phases: an initial phase of accelerated bone loss owing mainly to increased osteoclast-mediated bone resorption; followed by a slow but continuous phase of qualitative and quantitative bone loss as a result of the compromised function of both osteoblasts and osteocytes. While all skeletal cell types – namely osteoblasts, osteocytes and osteoclasts – are targeted by GCs, it is now understood that cells of the osteoblast lineage are the main effectors of GC-induced bone loss and the GC-induced rise in fracture risk.

Here we review the molecular and cellular targets of therapeutic doses of GCs with a particular focus on osteoblasts and osteocytes as well as the implications for clinical therapy of GIO.

## The Osteoblast Lineage as a Key Target for Excess GCs

**Figure 1:**
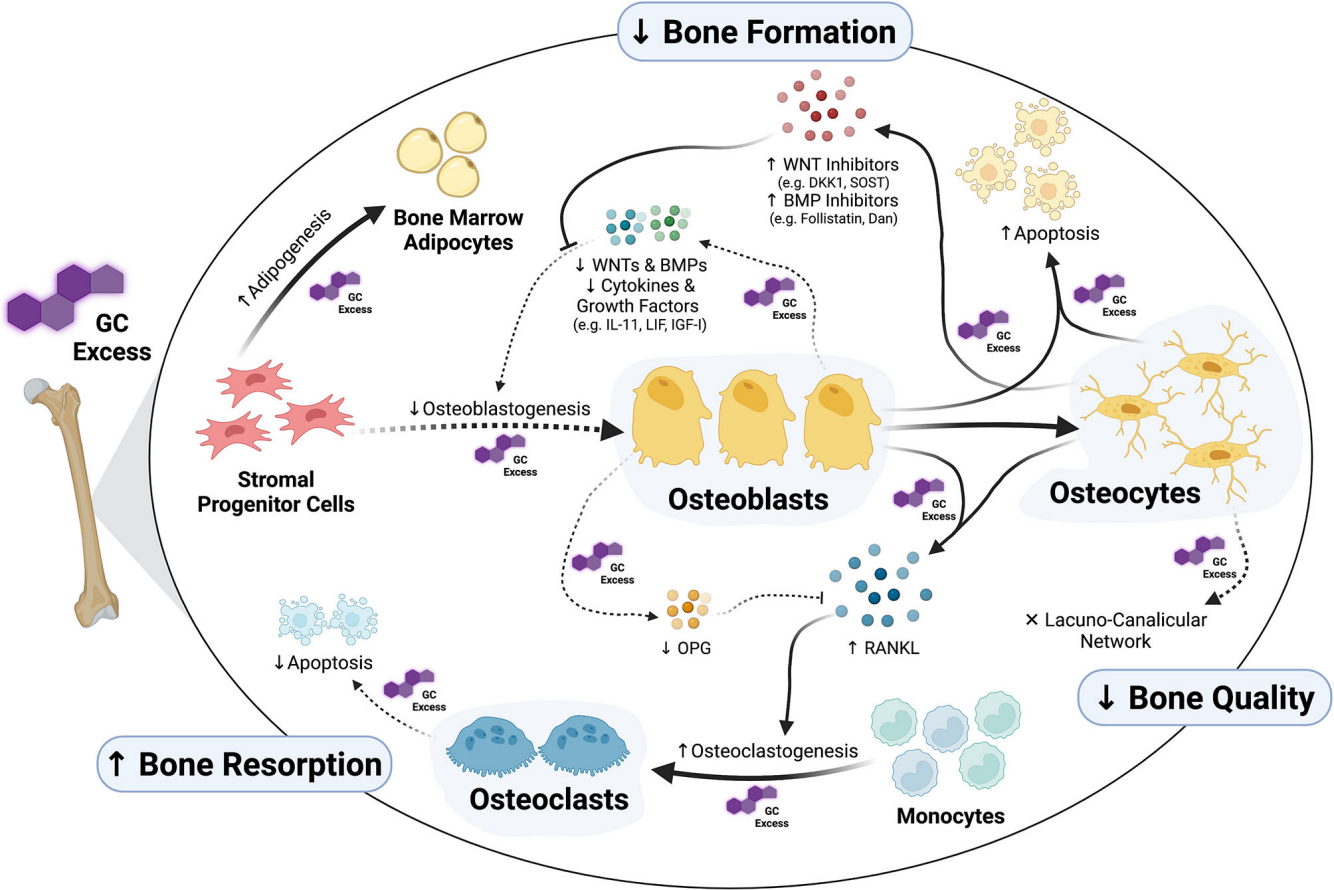
Osteoblasts and osteocytes as main targets of glucocorticoid (GC) excess in the skeleton. Exposure to supra-physiological levels of GCs affects many aspects of osteoblast formation and function. Whereas GCs inhibit osteogenic commitment of stromal progenitor cells by diversion into adipogenesis, they inhibit proliferation and differentiation of pre-osteoblasts through direct as well as autocrine/paracrine effects. Together with suppression of osteoblast function, all these GC-induced alterations in osteoblasts suppress bone formation. Additionally, GCs induce apoptosis of both osteoblasts and osteocytes and cause disruptions in osteocytic lacuna-canalicular network affecting bone quality. Osteoclast-mediated bone resorption is affected by GCs as well, especially through the regulation of the RANKL/OPG system *via* osteoblasts and osteocytes. The figure was created with BioRender.com.

Skeletal cells continually interact with one another through the process of bone remodeling. Bone remodeling includes the coordinated processes of bone formation and bone resorption. Formation of new bone is performed by osteoblasts, whereas bone resorption is carried out by osteoclasts. Osteocytes act as mechanosensors and orchestrate the skeletal remodeling process by initiating and governing the remodeling cycle (31, 32). While exogenous GCs affect all cells of the remodeling process – either directly or indirectly (**Figure 1**) –, cells of the osteoblast lineage, and therefore bone formation, are key targets of GCs in the skeleton.

Generally, exposure to supra-physiological levels of GCs results in a strong suppression of bone formation and the anabolic function of osteoblasts in both humans and rodents. Treatment of patients with therapeutic doses of GCs rapidly suppresses serum markers of bone formation such as osteocalcin, bone-specific alkaline phosphatase (ALP) and procollagen type I N-terminal propeptide (P1NP) (33–40). Similarly, prolonged exposure of rodents to excess GCs decreases the systemic markers of bone formation and the osteoblasts’ anabolic function, such as osteocalcin and P1NP (17, 41–46). Histomorphological analysis of bones from GC-treated rodents confirms these findings and reveals compromised bone formation and mineralization as well as a reduction in the number and surface of osteoblasts (17, 23, 43, 45, 47–49). Similar effects were observed in bone biopsies from GC-treated patients (50–53). Overall, GIO occurs in both rodents and humans with similar cellular and molecular features. Thus, rodents may act as a suitable model organism to investigate the molecular and cellular mechanism underlying GIO (54).

The significance of osteoblasts in the pathogenesis of GIO has been made clear through the utilization of genetically modified mouse models, in which GC-GR signaling has been disrupted in a cell-specific fashion. Protection of osteoblasts from excessive GC signaling by osteoblast-specific overexpression of the GC-inactivating enzyme, 11β-hydroxysteroid dehydrogenase type 2 (11β-HSD2), not only prevented GC-induced osteoblast apoptosis but also preserved osteoblast function and bone formation (43, 55). Similarly, specific deletion of GR in osteoblasts prevented both GC-driven bone loss as well as compromised bone formation (23). Some – though not all – studies investigating the disruption of GC signaling in osteoblasts/osteocytes during GC excess showed that not only osteoblast function and bone formation were preserved in this setting but also the GC-induced increase in osteoclast number and activity was prevented (43). Collectively, these results suggest that the adverse skeletal effects of exogenous GCs result to a large degree from their detrimental action on cells of the osteoblast lineage. Quantifying the overall contribution of osteoclasts to the development of GC-induced osteoporosis remains challenging. The selective abrogation of GC-GR signaling in osteoclasts (by GR knock-out) resulted in preserved bone resorption and preserved bone formation, indicating a prominent role for osteoclasts in GC-induced bone loss (56). However, – in the hands of different researchers – the osteoclast-specific disruption of GCs (either by 11β-HSD2 overexpression or conditional GR knockout) had no discernible protective effects against GC-induced bone loss since osteoblasts were readily affected by excess GCs (23, 57). Collectively, the weight of the evidence strongly points to the osteoblast lineage as a more impactful target of GCs in the skeleton compared to the cells of the osteoclast lineage.

## The Effects of GC Excess on the Formation and Function of Osteoblasts

GCs cause alterations in the formation and apoptosis of osteoblasts as well as their function, all of which contribute to the pathogenesis of GIO. *In vivo* and *in vitro* studies have determined that supra-physiological levels of GCs exert their deleterious effects on cells of the osteoblast lineage at all stages of differentiation, leading to reduced osteoblast formation. Moreover, GCs limit both function and lifespan of osteoblasts, ultimately resulting in compromised bone formation. Furthermore, through the intrinsic link between bone formation and bone resorption, GCs may alter osteoblast activity and function through their action in osteoblasts and osteocytes. The effects of exogenous GCs on molecular pathways within osteoblasts are manifold and the relative contribution of each identified pathway is not always quantifiable. Nevertheless, the main effects of GCs on osteoblasts can be outlined as follows:

### a) Decreased Osteogenic Cell Fate of Stromal Progenitor Cells

Given the multipotent nature of stromal progenitor cells in the bone marrow, supra-physiological levels of GCs induce diversion of these stem cells away from the osteoblast lineage towards the adipocyte lineage. Ultimately, this diversion of stem cell commitment leads to a decrease in the pool of osteoblast progenitors and limits bone formation. Accordingly, it has been shown that exposure to exogenous GCs in humans and rodents is associated with increased bone marrow adiposity (58–60). In line with these results, gene expression profiling of bone tissue from GC-treated mice displayed an induction of adipogenesis-related genes whereas osteogenic genes were downregulated (49). Moreover, bone marrow stromal progenitor cells from GC-treated rodents displayed reduced osteoblastogenesis *ex vivo* (23, 45, 48), with enhanced direction towards adipogenesis even in osteogenic media (59, 60). Similarly, exposure of bone marrow stromal progenitor cells to pharmacological levels of GCs results in decreased expression of essential osteogenic transcription factors such as runt-related transcription factor 2 (RUNX2), accompanied by concurrent increased expression of adipogenic transcription factors such as peroxisome proliferator- activated receptor gamma (PPARγ) and CCAAT-enhancer-binding protein alpha (C/EBPα) (61–66).

### b) Suppressed Proliferation of Osteoprogenitors

Acting also on committed osteoblast precursors, GCs have been shown to inhibit and suppress their proliferation prior to full differentiation. In pre-osteoblast cultures, exposure to pharmacological ‘micromolar’ concentrations of GCs was associated with cell cycle arrest at the G1 phase due to downregulation of cell cycle activators such as Cyclin A, Cyclin D, Cyclin-dependent kinase 2 (CDK2), CDK4 and CDK6 (67–70) as well as upregulation of cell cycle inhibitors such as p53, p21 and p27 (67, 69, 71). In addition, GCs were shown to suppress the proliferation of osteoblast precursors through suppression of intracellular mitogenic signaling pathways, such as mitogen-activated protein kinase (MAPK) signaling *via* a rapid increase in the expression of a tyrosine phosphatase, MAPK phosphatase 1/dual specificity protein phosphatase 1 (MKP1/DUSP1), leading to dephosphorylation of extracellular-signal-regulated kinases (ERK), p38 and c-Jun N-terminal kinase (JNK) (72–74). Interestingly, while non-specific tyrosine phosphatase inhibition reversed GC-induced suppression of pre-osteoblasts *in vitro* and partly prevented deleterious bone effects (of GCs) in a rat model of GIO, *Mkp1* knockout mice were not protected against the adverse effects of methylprednisolone treatment (72–76). In a different study *Mkp1* deletion was shown to exacerbate inflammatory bone loss (77). These results suggest that targeting MKP1 may not represent a viable strategy for the prevention of GC-driven bone loss.

### c) Inhibited Differentiation of Osteoblast Precursors Into Mature Osteoblasts

GC-induced inhibition of osteoblastogenesis is mediated mainly *via* suppression of signaling pathways involved in promoting osteoblast differentiation, importantly WNT and bone morphogenetic protein (BMP) pathways. First, GCs have been shown to inhibit the production of autocrine/paracrine WNT proteins, such as WNT7b, WNT10 and WNT16 (22, 78), as well as BMP proteins, such as BMP2, from mature osteoblasts (79–82). Conversely, the GC-driven suppression of osteoblast differentiation *in vitro* was corrected by supplementation of culture media with WNT and BMP proteins. Second, GCs increase the expression of inhibitory factors of the WNT and BMP signaling pathways from osteoblasts as well as osteocytes including WNT antagonists such as dickkopf1 (DKK1), sclerostin (SOST), secreted frizzled-related protein 1 (sRFP1) and axin-2 (22, 41, 49, 79, 83–89), as well as BMP antagonists, such as Follistatin and Dan (63, 79, 90). Third, exposure of pre-osteoblasts to supra-physiological levels of GCs suppresses the canonical WNT pathway through inducing degradation and inactivation of β-catenin, therefore inhibiting osteoblastogenesis (68, 91, 92). Moreover, suppression of growth factor pathways, such as insulin-like growth factor I (IGF-I), may contribute to the suppressive effects of GCs on osteoblastogenesis (93–96). GCs also suppress anabolic cytokines such as interleukin-11 (IL-11) and leukemia inhibitory factor (LIF) thereby reducing Janus kinase 2 (JAK2) – signal transducer and activator of transcription 3 (STAT3) signaling *via* inducing interaction of the monomeric glucocorticoid receptor with the transcription factor AP-1 (23, 97). Not only did supplementation of GC-treated osteoblasts with IL-11 (23, 97) and LIF (98) reverse the suppression in STAT3 signaling and osteoblast differentiation *in vitro*, treatment with LIF protected mice against GC-driven bone loss (98). Interestingly, reduced IL-11 expression was observed in other models of bone loss such as age-related suppression of bone formation, suggesting that IL-11 may be generally implicated in bone diseases (99, 100). Nevertheless, IL-11 is known to affect osteoclasts as well (101). Beside the direct targeting of key bone-anabolic pathways such as WNT and BMP signaling, GCs modulate the expression of miRNAs, including miR-29a, miR-34a-5p and miR-199a-5p, which regulate proliferation and differentiation of osteoblasts (102). A study by Wang and colleagues showed an association of GC-induced osteoporosis with miR-29a in rats, as GCs reduced the levels of miR-29a leading to a subsequent increase in deacetylation and ubiquitinylation of β-catenin, thus attenuating the pro-osteogenic impact of WNT signaling on differentiation of osteoblasts (103, 104). However, osteoblast-selective deletion of *Dicer*, an important enzyme in miRNA biogenesis, did not affect GC-induced suppression of osteogenesis both *in vitro* and *in vivo* (105).

### d) Decreased Function of Osteoblasts

In addition to suppressed osteoblast formation, GCs decrease the anabolic function of osteoblasts, i.e., secretion of osteoid matrix proteins (e.g., collagen and osteocalcin) and subsequent mineralization of the matrix itself. For instance, GCs downregulate *OCN* (the gene encoding osteocalcin) gene expression in human and rat osteoblasts through direct binding of the GC-GR complex to a negative GC-response element (-GRE) in the enhancer region of the osteocalcin gene leading to trans-repression (106–108). Also, the expression of collagen from osteoblasts was shown to be suppressed by excess GCs *via* transcriptional and post-transcriptional mechanisms (109, 110). Apart from the synthesis of bone matrix proteins, supra-physiological levels of GCs were shown to provoke matrix degradation through upregulating expression of metalloproteinases such as matrix metalloproteinase 13 (MMP13) from osteoblasts (49, 111).

## The Effects of Excess GCs on the Lifespan of Osteoblasts and Osteocytes

Aside from suppression of osteoblast differentiation and activity, exposure to pharmacological levels of GCs triggers apoptosis in osteoblasts as well as their descendants, osteocytes, limiting their lifespan. Apoptotic osteoblasts and osteocytes were clearly detectable in the bones not only from GC-treated rodents (17, 45, 48, 55, 112) but also from patients undergoing therapy with GCs (45, 52, 113). It may be inferred that the GC-induced osteoblast apoptosis, similarly to suppressed osteoblast differentiation, likely contributes to the compromised bone formation, ultimately leading to GC-induced loss of bone mass and increase in fracture risk. More importantly, prevention of GC-driven apoptosis in osteoblasts and osteocytes has been associated with preservation of bone mass as well as strength in mouse models of GIO. For instance, co-treatment of mice with bisphosphonates (48, 114), intermittent parathyroid hormone (PTH) (115) or osteoprotegerin (OPG) (116) alleviated the adverse effects of pharmacological GCs on osteoblast and osteocyte apoptosis as well as bone formation and mineralization resulting in protection from bone loss.

Despite the evidence outlined above, some studies failed to detect a GC-induced increase in apoptosis of osteoblasts and osteocytes despite the detrimental effects of GCs on bone formation (23). This might be related to differences in the mouse strain and/or the dose of GCs utilized in the study. Importantly, the induction of apoptosis in osteocytes and osteoblasts has been shown to be dose- and time-dependent. In response to low ‘nanomolar’ concentrations of GCs, osteocytes and osteoblasts rely on autophagy to repair cellular damage and maintain viability (112, 117–120). In mice treated with low dose GCs, an upregulation of the expression of anti-oxidant and autophagy genes as well as an appearance of autophagic osteocytes and osteoblasts was observed in the skeleton (112, 119). However, prolonged exposure and/or high ‘micromolar’ doses of GCs result in suppression of autophagy as well as excessive intracellular damage due to accumulation of autophagosomes inside osteocytes and osteoblasts, which ultimately lead to the activation of pro-apoptotic pathways and programmed cell death (112, 119, 121). Induction of autophagy in osteocytes and osteoblasts has been hypothesized to underpin a protective mechanism to preserve cellular viability (120, 122, 123); however, prolonged exposure to GCs is associated with suppressed autophagy leading to apoptosis (117, 123, 124). Indeed, enhancing autophagy *in vivo* by administration of the phytoecdysteroid, β-ecdysone, to GC-treated mice prevents GC-induced bone loss by reversing the suppression of bone formation and the induction of apoptosis in osteoblasts and osteocytes (121, 124). Likewise, pharmacological inhibition of autophagy was associated with an increase in GC-induced osteoblast apoptosis *in vitro* (117, 120). Nevertheless, the significance of autophagy in the detrimental effect of GCs on cells of the osteoblast lineage remains overwhelming (122, 125). Targeting apoptosis and autophagy of osteoblasts and osteocytes has been highlighted as a therapy for not only GC-driven bone loss (125), but also in age-related osteoporosis (126, 127).

Several studies using *in vitro* osteoblast and osteocyte cultures revealed some of the molecular mechanisms underpinning GC-induced apoptosis. Not only mechanisms related to regulation of transcription, but also rapid non-genomic mechanisms have been attributed to the apoptotic impact of GCs on the osteoblast lineage. The most evident subcellular apoptotic pathways in osteoblasts and/or osteocytes influenced by genomic GR actions have been upregulation of pro-apoptotic proteins such as BIM, BAK, p53 and p21 (67, 71, 128–130), as well as the suppression of survival, anti-apoptotic factors such as BCL-2, BCL-Xl and MCL-1 (67, 112, 131, 132). In addition, suppression of MAPK – ERK pathway through upregulation of MKP1/DUSP1 may act as another mechanism for GC-driven apoptosis in osteocytes and osteoblasts, as a non-selective protein tyrosine inhibitor was able to prevent GC-driven osteoblast apoptosis *in vitro* and *in vivo* (133). An increase in oxidative stress in the endoplasmic reticulum (ER) is one of the non-genomic pathways implicated in accumulation of reactive oxygen species (ROS), which may activate JNK signaling and programmed cell death in osteoblasts (84, 131, 134–136). Generally, prevention of oxidative stress exerts protective effects on osteoblasts and osteocytes thus preserving bone formation in addition to mediating anti-resorptive effects on osteoclasts (137). Prevention of ER stress and ROS accumulation *via* knocking down *Eif2a* (Eukaryotic Translation Initiation Factor 2A) not only prevented GC-induced apoptosis *in vitro* and *in vivo*, but also was associated with protection against bone loss (138). Inducing the protein tyrosine kinase 2 beta (PYK2) pathway and blocking focal adhesion kinase (FAK) signaling may contribute to GC-induced apoptosis in cells of the osteoblast lineage (136). In a recent report, genetic and pharmacological inactivation of Pyk2 signaling was proven effective in preventing not only apoptosis in osteoblasts and osteocytes, but also GC-induced bone loss, although reversing compromised osteoclast function was shown to likely contribute to such protective effects (139). Moreover, induction of Fas receptor/CD95 may advance apoptotic pathways in osteoblasts and osteocytes (140). Two recent studies hypothesized that long-non coding (lnc) RNAs are involved in GC-induced osteoblast apoptosis. Long-non coding RNAs are a large family of RNA molecules that are able to regulate protein expression and/or function. Lnc-MALAT1 and lnc-EPIC1 expression were shown to be altered in human osteoblasts treated with dexamethasone and to interact with AMP-activated protein kinase signaling and MYC [a regulator of osteoblast survival] (141, 142). However, the role of lncRNA in GIO remains to be validated *in vivo*.

## The Effects of Excess GCs on the Function of Osteocytes

Osteocytes play a crucial role in bone homeostasis through modulating the formation and activity of osteoblasts and bone formation *via* the release of WNT signaling inhibitors, sclerostin and dickkopf1 (DKK1) (143). In a number of studies, an upregulation of sclerostin gene and protein expression has been observed in the cortical-rich bones from GC-treated mice, where osteocytes are generally more abundant than osteoblasts (39, 49, 87, 144). Strong evidence for the significant contribution of the GC-driven upregulation of sclerostin in osteocytes to GIO has come from studies of abrogated sclerostin action in rodent models of excess GCs. Administration of anti-sclerostin antibodies to rats and mice prevented the development of GC-induced bone loss largely *via* preserving the function and number of osteoblasts and maintaining bone formation and mineralization (46, 145). In addition, knocking out *Sost* (the gene encoding sclerostin) in mice provided protection from GC-driven bone loss (144). In humans, the contribution of sclerostin to GC-induced bone loss is less clear. One study described a trend increase in serum levels of sclerostin in patients receiving pharmacological GCs (36). However, the serum levels of sclerostin were decreased in the patients treated with GCs in comparison to matched controls (39), and similar results were observed after acute treatment with therapeutic GCs in another study (146). DKK1, another WNT inhibitor expressed in osteocytes, is upregulated in GC-treated animals, and anti-sense silencing of *Dkk1* in mice was effective in preserving bone mass as well as bone formation during GC excess (49, 89). In a recent study, conditional knockout of *Dkk1* in osteoblasts and/or osteocytes prevented the development of GC-induced bone loss *via* reversing the adverse effects of GCs on osteoblasts and bone formation (41). Notably, both sclerostin and DKK1 have emerged as promising therapeutic targets in a number of bone diseases (147), and may be utilized clinically for the management of GIO in the future.

Aside from affecting the regulatory role of osteocytes through sclerostin and DKK1, several alterations in the bone environment around the osteocyte-lacunar environment have been reported in response to pharmacological levels of GCs. In bones from GC-treated mice, changes in the bone matrix surrounding osteocyte lacunae were observed, specifically an increased lacunae size as well as perilacunar hypomineralization (17). Additionally, these effects were associated with compromised bone strength (17). Moreover, osteocyte perilacunar remodeling was shown to be adversely affected by exogenous GCs: a GC-induced suppression of the expression of matrix metalloproteinases (MMPs) leads to collagen disorganization and degeneration of the lacuno-canalicular network (148). In the *in vitro* setting, Gao et al. were able to show that the gap-junction connectivity of osteocytes was adversely affected by dexamethasone treatment of an osteocyte cell line (MLYO-cells). These dexamethasone-induced changes resulted in a suppressed amount of Connexin 43 due to degradation by autophagy, thus leading to shortening of osteocyte dendrites, which likely contributes to the compromised connectivity between osteocytes (149). Furthermore, GCs were shown to impair the skeletal vasculature leading to a reduction in solute transport from the circulation to the osteocyte-lacunar-canalicular network and a decrease in the interstitial fluid, thereby compromising bone strength (150). Interestingly, PTH treatment was able to rescue skeletal vascularity during GC exposure (151). More recently, two studies highlighted the role of the skeletal vasculature in the context of GCs during growth. GC-exposure in young mice (typically around 3 weeks of age) impaired angiogenesis and osteogenesis simultaneously (152, 153). Liu et al. were able to show that osteoclast-derived angiogenin was decreased in response to elevated levels of GCs, leading to an increase in blood vessel senescence (153).

In summary, GCs exert a detrimental impact on the function and lifespan of osteocytes leading not only to compromised bone formation but also to disruptions in the lacunar-canalicular network (**Figure 1**). The GC-induced dysfunction of the osteocyte-canalicular network may represent a potential mechanism underlying the predisposition to developing fractures shortly after initiation of GC treatment prior to any significant decreases in BMD – a frequent clinical observation (8). The role of the skeletal vasculature in GIO has been highlighted through recent studies and its role needs further exploration – particularly its connection to bone cells (i.e. osteoblast, osteocytes and osteoclasts) as well as its link to fracture risk.

## The Effects of GC Excess on Osteoclasts

While the adverse effects of GCs on osteoblasts and osteocytes contribute to the long-term phase of bone loss and compromised bone strength in GIO, the initial rapid phase of bone loss typically observed in humans and rodents originates from a rapid induction of osteoclast-mediated bone resorption. In a number of *in vivo* studies, treatment of rodents with GCs results in a rapid elevation of systemic parameters of bone resorption including serum and/or urinary bone resorption markers, such as carboxy-terminal collagen crosslinks (CTX) and tartrate-resistant acid phosphatase-5b (TRAP-5b), upon exposure to supra-physiological levels of GCs (17, 41, 43, 46, 49). In addition, in the bones from GC-treated rodents, an increase in the number of osteoclasts, as well as an increase in gene expression of osteoclast-mediated bone resorption have been reported shortly after exposure to exogenous GCs (17, 45, 46, 48, 49). While some studies also showed upregulation of osteoclast activity and bone resorption markers at later time-points (41, 47, 154, 155), other studies failed to detect increases in bone resorption especially after prolonged GC exposure (45, 156). In addition, one study by Henneicke et al. showed that treatment with corticosterone affected osteoclasts in a site-specific manner in rodents: an increase in osteoclasts was detected in the endocortex, while they were reduced in the pericortex of tibia from GC-treated mice (43).

Several *in vivo* and *in vitro* studies have determined that the mechanisms of elevated osteoclast-mediated bone resorption in GIO originate not only from direct effects of GCs in osteoclasts, but also from indirect effects *via* the osteoblast lineage. It has been shown that the early increase in osteoclastic bone resorption may be accounted for by an increase in the survival of mature osteoclasts and reduced predisposition to apoptosis (48, 56, 57, 157). However, the direct impact of excess GCs on osteoclastogenesis and osteoclast activity has been controversially discussed due to conflicting results from *in vitro* studies. While some authors observed that pharmacological GCs augmented osteoclast formation and resorptive activity (158–160), others reported a reduction in proliferation of osteoclast precursors (56, 157). Additionally, bone marrow macrophages (osteoclast precursors) from GC-treated animals gave rise to a lower number of osteoclast precursors *ex vivo* than their placebo controls (45, 48). Furthermore, exposure of *in vitro*-formed osteoclasts to GCs increased their longevity, yet, in the same study, it decreased their resorptive function due to defects in cytoskeleton reorganization (56, 157). Interestingly, a recent study found that dexamethasone delayed the formation of multinucleated osteoclasts on plastic surfaces yet increased the formation of resorption pits on dentin slides (161). Ultimately, the contribution of direct effects of GCs on osteoclasts to the overall phenotype of GIO remains unclear due to the large amount of conflicting data.

In contrast, the indirect effects of GC excess on osteoclastogenesis and bone resorption have been well characterized across both *in vivo* and *vitro* studies. The receptor activator of NF-κB ligand (RANKL) – osteoprotegerin (OPG) system, which plays a crucial role in the differentiation of osteoclasts, is affected to a large degree by pharmacological levels of GCs. Several studies demonstrated that supraphysiological levels of GCs induce the expression and production of RANKL from osteoblasts in culture (162–165), a finding also confirmed *in vivo* (144, 166). Administration of a human anti-RANKL antibody to mice expressing human RANKL conferred protection from GC-induced bone loss (166). Some studies suggest that osteocytes – rather than osteoblasts – are the principle source of RANKL *in vivo* (167, 168); however, a more recent study failed to show an increase in RANKL in the osteocyte-enriched bones from GC-treated rodents (47). Interestingly, in the same study a genetic knockdown of *Rankl* specifically in osteocytes provided partial protection from GC-induced bone loss *via* reversal of the osteoclast induction (47).

Aside from RANKL, GCs have been shown to reduce the production of OPG, the decoy receptor of RANKL, from osteoblasts and/or osteocytes, which may aide GC-driven osteoclastogenesis (47, 144, 162–165, 169, 170). Additionally, administration of OPG was able to reduce GC-induced bone resorption in calvarial organ culture (165) as well as prevent GC-induced bone loss in rodents (116). Indeed, some studies suggest that the increase in the ratio between RANKL and OPG in bone may be largely due to suppressed OPG rather than due to increased RANKL (47, 144, 169). Other indirect contributors to GC-induced bone resorption include macrophage colony-stimulating factor (M-CSF): exposure of osteoblasts to pharmacological levels of GCs was shown to induce the expression of M-CSF, which acts as an essential factor for osteoclast differentiation (171).

In summary, GCs certainly exert direct effects on osteoclasts; however, whether these direct effects contribute to the phenotype of GC-induced bone loss remains controversial. In contrast, *in vivo* and *in vitro* studies clearly demonstrate that GCs readily induce osteoclast formation indirectly through upregulation of pro-osteoclastogenic factors derived from cells of the osteoblast lineage (**Figure 1**).

## Targeting Osteoblasts as a Therapeutic Approach for the Management of GIO

As the mainstay of osteoporosis therapy anti-resorptive bisphosphonates have been widely used in the therapy of GIO. Generally, the use of bisphosphonate in GIO leads to an increase in bone mineral density compared to placebo or calcium and vitamin D supplements (15). Thus, three different bisphosphonates are currently approved for the treatment of GIO, namely risedronate (172, 173), alendronate (174) and zoledronic acid (175). Zoledronic acid has been shown to be superior to risedronate in GIO and postmenopausal osteoporosis (175) and is generally considered the most potent bisphosphonate. Although not an osteoanabolic therapy, denosumab, a RANKL inhibitor, counteracts a key mechanism of GCs in bone – the induction of RANKL release from osteoblasts and osteocytes. Clinical studies showed a larger increase in bone mineral density (BMD) during denosumab therapy compared to risedronate confirming its superiority to one of the bisphosphonates in GIO (176, 177). Unfortunately, denosumab has not yet been evaluated against the most potent bisphosphonate zoledronic acid in the context of GC use, but its value in the treatment of GIO is undeniable.

While bisphosphonates and denosumab have been successfully utilized to combat GIO, they only offset the GC-induced activation of osteoclasts – which is of particular importance during the initial stage of GC-therapy. However, as outlined above, bisphosphonates fail to address the suppression of osteoblast and osteocyte function, which are a crucial part of the pathogenesis of GIO. The development of targeted osteoporosis therapies opens up the possibility of targeting the mechanism underlying GIO more specifically.

Currently only one osteoanabolic agent, targeting bone formation directly, is approved for the treatment of GIO. As a parathyroid hormone (PTH) analog (1-34 PTH), teriparatide primarily stimulates bone formation – even though bone resorption is activated in response to teriparatide as well. However, bone resorption is initiated much later than bone formation resulting in an ‘anabolic window’, during which new bone is formed (178). Mechanistically, as an anabolic therapy it mitigates the GC-induced suppression of osteoblast (and osteocyte) activity, which forms a key part of the mechanism underpinning GIO. In the clinical setting, teriparatide has been shown to increase BMD to a larger extent than risedronate (179) and alendronate (180, 181) during GC exposure, thus highlighting the key role of osteoanabolic therapy for GIO. At this stage, no adequate comparison between teriparatide and denosumab exists during GIO (182), hence, no conclusions may be drawn regarding their relative potency in the context of GC therapy.

Novel osteoanabolic therapies such as the PTH-related protein analogue abaloparatide (183) and the anti-sclerostin antibody romosozumab (184, 185), which have been approved for the use in postmenopausal osteoporosis, have not yet been evaluated in GIO. Given their osteoanabolic properties, they may prove similarly effective as teriparatide.

In summary, all available pharmacological therapies are effective in GIO, this includes bisphosphonates, denosumab as well as teriparatide (**Table 1**). Therapies, which target the molecular and cellular mechanisms of GCs in the skeleton such as denosumab and teriparatide, have been shown to be superior to bisphosphonates in GIO. Some (186) but not all (187) guidelines reflect this by recommending the use of teriparatide in severe cases of GIO or following the occurrence of fractures under treatment with bisphosphonates.

**Table 1:** Current and future pharmacological GIO therapy.

Drug	Administration	Mechanism of action	Renal function	Approval for GIO
Risedronate	oral, 5 mg daily or 35 mg weekly	anti-resorptive (bisphosphonate)	avoid if GFR < 50 (35) mL/min/1.73	yes
Alendronate	oral, 70 mg weekly	anti-resorptive (bisphosphonate)	avoid if GFR < 50 (35) mL/min/1.73	yes
Zoledronic acid	i.v., 5 mg every 12 months	anti-resorptive (bisphosphonate)	avoid if GFR < 50 (35) mL/min/1.73	yes
Denosumab	s.c., 60 mg every 6 months	anti-resorptive (RANKL antibody)	no adjustment	yes
Teriparatide	s.c., 20 µg daily	osteo-anabolic [recombinant PTH (1-34)]	no adjustment	yes
Abaloparatide	s.c., 80 µg daily	osteo-anabolic [recombinant PTH (1-34)]	no adjustment	no
Romosozumab	s.c., 210 mg every month	osteo-anabolic (synthetic PTHrp analog)	no adjustment	no

(Of note, in addition to calcium and vitamin D supplementation).

## Summary

Glucocorticoids affect the three main cell types within the skeleton – osteoblasts, osteocytes and osteoclasts – ultimately leading to a loss of bone mass and bone quality as well as causing a substantial increase in fracture risk. Preclinical studies have highlighted the key role of osteoblasts and osteocytes in the pathogenesis of glucocorticoid-induced osteoporosis and emerging clinical evidence supports the superiority of osteoblast-targeted therapies. Future studies should develop and evaluate therapeutic strategies that not only alleviate GC-induced bone resorption but also prevent the GC-induced damage to osteoblasts and osteocytes and activate bone formation. Furthermore, novel aspects of GIO such as the role of the skeletal vasculature ought to be explored in greater detail.

BOX 1 **Bone mineral density as a surrogate parameter in GIO.**

GCs have been shown to substantially increase fracture risk in humans. Interestingly, the increase in fracture risk manifest itself immediately after the commencement of GC therapy (8), leading to the hypothesis that GCs may damage bone beyond the loss of bone mass. And indeed, studies were able to establish that in patients suffering from GIO fractures occurred more frequently compared to patients with postmenopausal osteoporosis even when BMD scores were taken into account (13). Similarly, it has been established that the commonly used FRAX algorithm underestimates the occurrence of fractures in subjects treated with GCs (15). More recently the use of trabecular bone score (TBS) has been shown to potentially remedy some of these concerns (16); however, its use has not been widely adopted and/or established as a diagnostic tool in GIO. Overall, the predictive value of BMD is reduced in GIO compared to postmenopausal osteoporosis. This is of particular concern as virtually all studies assessing the use of anti-osteoporotic medication in GIO utilize BMD as a surrogate parameter for fractures. Studies were not adequately powered to allow for an analysis of fracture risk. This should be taken into account when evaluating the results of clinical trials comparing therapeutic agents in the context of GIO.

Preclinical studies have attempted to assess the underlying reason for the particularly high fracture risk in GIO compared to postmenopausal osteoporosis. Studies in rodents were able to link the high fracture risk in GIO as well as the rapid onset of fractures following commencement of GC-therapy to their detrimental effects on osteocytes. Lane et al. highlighted the role of the lacunar-canalicular network in this context, which is largely maintained by osteocytes (17). Others have built on this idea and highlighted the role of the skeletal vasculature in GIO, see section ‘*The Effects of Excess GCs on the Function of Osteocytes’* for further details. However, the rapid increase in fracture risk with commencement of GC-therapy may also be the result of systemic effects of supraphysiological levels of GCs; i.e. GCs may decrease muscle strength and adversely affect coordination and/or lead to an increase in falls (and thus fractures) due to their effects in the central nervous system. Hence, whether the rapid and strong increase in fractures following commencement of therapeutic GCs is a result of bone-intrinsic effects of GCs or GC-action elsewhere in the body remains to be determined.

## Author Contributions

All authors listed have made a substantial, direct, and intellectual contribution to the work, and approved it for publication.

## Funding

Deutsche Forschungsgemeinschaft (Grant numbers: BA-6428/1-1 and HE-8391/1-1) and Else Kröner-Fresenius-Stiftung.

## Conflict of Interest

HH has received travel support, speaking fees and honoraria for scientific consultation from Amgen, Takeda and Novo Nordisk. LH reports honoraria from Amgen, Alexion, Kyowa Kirin International, and UCB for scientific consultation to himself, and trial compensation from Ascendis, Novartis, and Takeda to his institution.

The remaining author declares that the research was conducted in the absence of any commercial or financial relationships that could be construed as a potential conflict of interest.

The handling editor declared a past collaboration with one of the authors LH.

## Publisher’s Note

All claims expressed in this article are solely those of the authors and do not necessarily represent those of their affiliated organizations, or those of the publisher, the editors and the reviewers. Any product that may be evaluated in this article, or claim that may be made by its manufacturer, is not guaranteed or endorsed by the publisher.

## Abbreviations

11β-HSD2, 11β-hydroxysteroid dehydrogenase type 2; AP-1, activator protein 1; ALP, alkaline phosphatase; BMP, bone morphogenic protein; BMD, bone mineral density; CTX, carboxy-terminal collagen crosslinks; C/EBPα, CCAAT-enhancer-binding protein alpha; CDK, Cyclin-dependent Kinase; DUSP1, Dual-specificity phosphatase 1; DKK1, dickkopf1; ER, endoplasmic reticulum; ERK, extracellular-signal-regulated kinases; Eif2a, Eukaryotic Translation Initiation Factor 2A; FAK, focal adhesion kinase; GR, glucocorticoid receptor; GCs, glucocorticoids; -GRE, negative GC-response element; GIO, GC-induced osteoporosis; GLOW, Global Longitudinal Study of Osteoporosis in Women; IL-11, interleukin-11; IGF-1, insulin-like growth factor I; JNK, c-Jun N-terminal kinase; JAK2, Janus kinase 2; LncRNAs, long non-coding RNAs; LIF, leukemia inhibitory factor; MKP1, MAPK phosphatase 1; MAPK, mitogen-activated protein kinase; MMP, matrix metalloproteinase; M-CSF, Macrophage colony-stimulating factor; NF-κB, nuclear factor kappa-light-chain-enhancer of activated B cells; OPG, osteoprotegerin; OCN, osteocalcin; P1NP, procollagen type I N-terminal propeptide; PPARγ, Peroxisome proliferator-activated receptor gamma; PTH, parathyroid hormone; PYK2, Protein-tyrosine kinase 2 beta; RUNX2, runt-related transcription factor 2; RANKL, receptor activator of nuclear factor kappa-B ligand; ROS, reactive oxygen species; SOST, sclerostin; sRFP1, Secreted frizzled-related protein; STAT3, Signal Transducer And Activator Of Transcription 3; TRAP-5b, tartrate-resistant acid phosphatase-5b; TBS, trabecular bone score.
